# Biocompatible Potato-Starch Electrolyte-Based Coplanar Gate-Type Artificial Synaptic Transistors on Paper Substrates

**DOI:** 10.3390/ijms232415901

**Published:** 2022-12-14

**Authors:** Hyun-Sik Choi, Young-Jun Lee, Hamin Park, Won-Ju Cho

**Affiliations:** 1 Departments of Electronic Materials Engineering, Kwangwoon University, Gwangun-ro 20, Nowon-gu, Seoul 01897, Republic of Korea; 2 Departments of Electronic Engineering, Kwangwoon University, Gwangun-ro 20, Nowon-gu, Seoul 01897, Republic of Korea

**Keywords:** synaptic transistors, potato starch, artificial neural networks, biocompatible, biodegradable

## Abstract

In this study, we propose the use of artificial synaptic transistors with coplanar-gate structures fabricated on paper substrates comprising biocompatible and low-cost potato-starch electrolyte and indium–gallium–zinc oxide (IGZO) channels. The electrical double layer (EDL) gating effect of potato-starch electrolytes enabled the emulation of biological synaptic plasticity. Frequency dependence measurements of capacitance using a metal-insulator-metal capacitor configuration showed a 1.27 μF/cm^2^ at a frequency of 10 Hz. Therefore, strong capacitive coupling was confirmed within the potato-starch electrolyte/IGZO channel interface owing to EDL formation because of internal proton migration. An electrical characteristics evaluation of the potato-starch EDL transistors through transfer and output curve resulted in counterclockwise hysteresis caused by proton migration in the electrolyte; the hysteresis window linearly increased with maximum gate voltage. A synaptic functionality evaluation with single-spike excitatory post-synaptic current (EPSC), paired-pulse facilitation (PPF), and multi-spike EPSC resulted in mimicking short-term synaptic plasticity and signal transmission in the biological neural network. Further, channel conductance modulation by repetitive presynaptic stimuli, comprising potentiation and depression pulses, enabled stable modulation of synaptic weights, thereby validating the long-term plasticity. Finally, recognition simulations on the Modified National Institute of Standards and Technology (MNIST) handwritten digit database yielded a 92% recognition rate, thereby demonstrating the applicability of the proposed synaptic device to the neuromorphic system.

## 1. Introduction

The continuously increasing data throughput in artificial intelligence and big data has increased the importance of efficient information processing [1,2]. However, the Von Neumann architecture computing system is plagued by problems such as high-power consumption and slow processing speed for large data because of the bottleneck caused by the separation of memory elements and processing units [3,4,5]. To solve this problem, a neuromorphic computing system that mimics the human brain has been developed. Owing to the human brain capability of simultaneously performing high-level consumption and storage with low power, neuromorphic computing systems facilitate large-scale parallelization and simultaneous computation and storage at low power [6,7,8]. It is an important biological function facilitating multiple operations in the human brain [9,10]. Synaptic plasticity, which is the strength of synaptic connections depending on the situation, represents the change in synaptic weight that is controlled by the movement of ions through the synapse. For an efficient brain-inspired neuromorphic computing system, artificially implementing synaptic plasticity is essential. Therefore, synaptic devices, which are electronic devices used for implementing synaptic plasticity, are crucial to efficient neuromorphic computing systems. Because neuromorphic systems combine storage and computation, in general synaptic devices have been developed from two-terminal nonvolatile memory (NVM) devices such as resistive random-access memory (RRAM) and phase change memory (PCM) [11,12,13]. Meanwhile, three-terminal synaptic devices based on field-effect transistor (FET) structures can simultaneously transmit information and complete learning functions. Consequently, they extend the device concept to artificial synaptic electronics and can perform more complex calculations, and is thus suitable for building high-performance neural networks [14,15,16]. There are many types of synaptic transistors. Among them, synaptic transistors with electrolyte gate insulators adjust channel conductivity through the migration of ions in the electrolyte [17,18,19]. In response to a gate voltage, mobile ions and electrons in the insulator and channel layers, respectively, are concentrated within a very short distance at the channel/electrolyte interface to form an electrical double layer (EDL). Since the formed EDL has a large capacitance due to a nanogap-level distance between two opposite charge groups, it is applicable to the gate insulator of the FET. Therefore, EDL transistors using EDL have features that can further reduce power consumption [20,21]. Currently, many studies are being conducted to utilize eco-friendly and biocompatible natural organic materials that are harmless to the human body, such as cellulose, chitosan, milk, and starch, for the manufacturing of EDL transistors [19,22,23,24]. These materials are inexpensive, abundant, and biocompatible, which can lower the manufacturing costs. In addition, the use of paper substrates rather than silicon or glass substrates offers advantages in terms of process cost and use of disposable electronics [25,26].

This study proposed a potato-starch electrolyte-based synaptic transistor on a paper substrate. Fourier transform infrared (FTIR) spectroscopy was used to analyze the chemical group and molecular structure of the potato-starch electrolyte membrane. Further, to verify the EDL gating effect, the frequency-dependent capacitance was measured using metal-insulator-metal (MIM) capacitors having an aluminum/potato-starch electrolyte/platinum configuration. The indium-tin oxide (ITO) gate and indium–gallium–zinc oxide (IGZO) channel for pre- and post-synapses were formed on the same plane to ensure a coplanar gate structure, with ions in the potato-starch electrolyte used as neurotransmitters. The electrical properties of the prepared coplanar gate-type potato-starch EDL transistor were evaluated through transfer and output characteristic curves measurements. In addition, excitatory post-synaptic current (EPSC), paired-pulse facilitation (PPF), frequency-dependent multi-spike EPSC, and potentiation/depression were measured to examine short- and long-term synaptic plasticity. Finally, to demonstrate the applicability of the proposed device to neuromorphic systems, a recognition simulation was performed on the Modified National Institute of Standards and Technology (MNIST) handwritten digit database based on a multi-layered artificial neural network.

## 2. Results and Discussion

### 2.1. Characteristics Verification of Potato Starch

**Figure 1:**
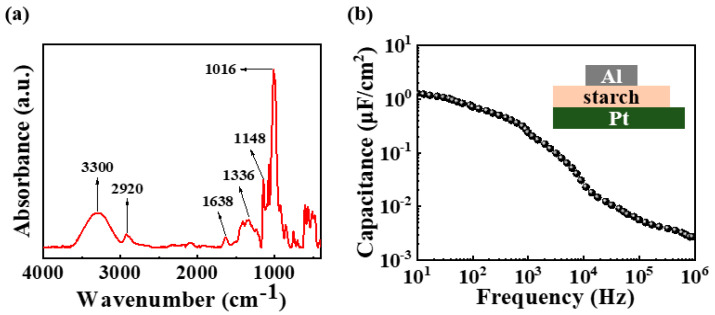
(**a**) FTIR spectrum of solid potato-starch electrolyte and (**b**) frequency-dependent capacitance of potato-starch electric double layer (EDL).

Prior to the application of the potato-starch electrolyte to the synaptic transistor, the chemical groups and molecular structures of the dried potato-starch electrolyte membrane were analyzed by FTIR. Figure 1a shows the FTIR spectrum of the solid electrolyte in the frequency range of 4000 to 900 cm^−1^. The peak at 3300 cm^−1^ was derived from the O-H stretching vibration, that at 2920 cm^−1^ indicated the stretching vibration of the C-H bond, and that at approximately 1638 cm^−1^ represented the angular deformation of water [24,27]. Further, the peaks at 1336 and 1016 cm^−1^ represent the modifications of the CH_2_, C-OH, and CH_2_ groups, respectively [24,27]. The presence of -OH groups can contribute to proton conductivity in electrolytes [24]. Specifically, mobile protons trigger ionic movements inside the gate insulator, which is fundamental to the synaptic behavior. When using an electrolyte material as an ion-based gate dielectric, ions in the electrolyte and electrons in the channel form an EDL with a nanogap between two oppositely charged layers in response to gate bias. Therefore, an EDL can be considered to be a parallel-plate capacitor. The EDL is formed with a very short thickness and exhibits a considerably large capacitance, which induces a strong coupling effect between the gate and channel [8,28]. Therefore, the EDL effect in potato-starch electrolytes controls ionic conductivity at low voltages, and consequently facilitates the implementation of efficient artificial synapses. Figure 1b shows the capacitance of an Al/potato-starch electrolyte/Pt-structured MIM capacitor at frequencies ranging from 10 Hz to 1 MHz. A high capacitance of 1.27 μF/cm^2^ was measured at a low frequency of 10 Hz; however, the capacitance decreased with increasing frequency, measuring 2.7 × 10^−3^ μF/cm^2^ at a high frequency of 1 MHz. This can be attributed to the response time of the ions in the electrolyte. At high frequencies, there is no ion accumulation at the interface because of the lack of response time. In contrast, at low frequencies, the ion response time is sufficient to form an ion layer at the interface, resulting in a high capacitance. The corresponding C-*f* curves demonstrate the EDL effect of the potato-starch electrolytes, indicating their applicability to synaptic transistors with a low driving voltage.

### 2.2. Electrical Properties and Synaptic Operation of Potato-Starch EDL Transistors

**Figure 2:**
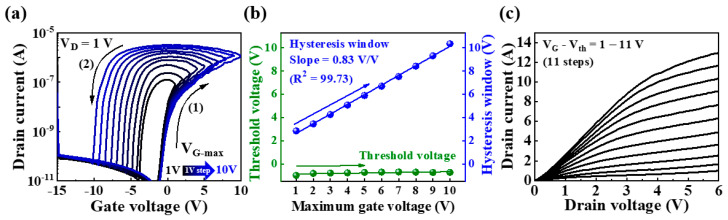
(**a**) Double-sweep transfer curves measured with V_G_max_ increasing in 1 V increments from 1 to 10 V; (**b**) threshold voltage and hysteresis window for V_G_max_; and (**c**) output characteristic curves of potato-starch EDL transistors.

Figure 2a shows that the changes in the double-sweep transfer curves measured as the maximum gate voltage (V_G_max_) was increased from 1 to 10 V in 1 V increments. Upon performing a forward sweep, protons in the electrolyte migrated to the electrolyte/IGZO channel interface; however, in case of a backward sweep, the protons slowly return to their original position, resulting in counterclockwise hysteresis. With increase in the applied V_G_max_, an increased number of protons migrated to the interface in proportion to V_G_max_, thereby increasing the relaxation time. Eventually, the hysteresis window increased linearly with V_G_max_. Figure 2b shows the threshold voltage (V_th_) and hysteresis window extracted from the transfer curves for V_G_max_. It is evident that V_th_ is fixed at −0.2 V regardless of V_G_max_, whereas the hysteresis window increased from 2.85 to 10.35 V with a gradient and linearity of 0.83 *v/v* and 99.73, respectively. Figure 2c shows the output characteristic curves measured at 1 V intervals from 0 to 6 V of the drain voltage (V_D_) while increasing V_G_–V_th_ from 1 to 11 V. With increase in V_D_, the drain current (I_D_) increased linearly and then gradually reached saturation. Further, with the increase in V_G_–V_th_, I_D_ increased, thus exhibiting the typical output characteristics of n-type FETs.

**Figure 3:**
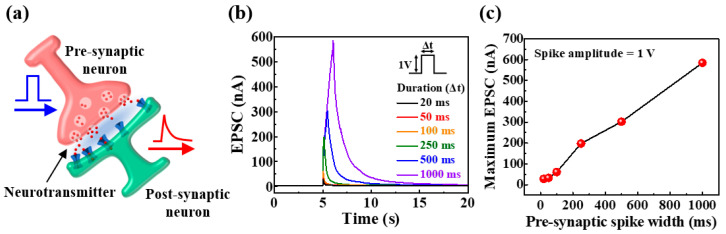
(**a**) Schematic of biological neuron and synapse; (**b**) excitatory post-synaptic currents (EPSCs) generated by pre-synaptic single-spike (1 V amplitude) for various durations (20 to 1000 ms); and (**c**) maximum EPSC as a function of pre-synaptic pulse duration.

Figure 3a shows a schematic of the signal transmission mechanism in a biological synapse. Upon stimulating the pre-synaptic neurons, the neurotransmitter moved through the synapse to the post-synaptic neuron, thus resulting in an EPSC [29]. In a nervous system, the concentration of neurotransmitters determines the synaptic connection strength [30]. In artificial synaptic devices, owing to the possibility of modulating EPSCs through the ion flux of electrolytes, the measured EPSCs are considered to be a basic expression of synaptic weight [31]. Synaptic plasticity is an experience-dependent mechanism for changing synaptic weights, and is the basis for learning and memory in the human brain [31]. Therefore, artificial synaptic devices should implement the working mechanisms of biological synapses in a similar manner. In the prepared potato-starch EDL transistors, the ITO coplanar gate and the IGZO channel serve as the pre- and post-synapse, respectively, and the mobile ions in the potato-starch electrolyte function as neurotransmitters. In addition, the EPSC was evaluated for presynaptic stimulation by measuring the channel current between ITO S/D. Figure 3b shows the pulse-dependent single-spike EPSCs for the application of a pulse with an amplitude of 1 V and duration of 20 to 1000 ms to the gate. It is evident that the EPSC increased with an increase in the presynaptic single-spike duration. Figure 3c shows the maximum EPSC values as a function of presynaptic spike width. The maximum EPSC was 28.5 nA at a pulse duration of 20 ms and continued to increase with pulse duration, increasing to 584.4 nA at 1000 ms. This is because the longer the spike stimulus applied to the pre-synapse, the greater the proton migration to the channel interface of the electrolyte, which increases the channel conductivity. Moreover, with an increase in the pulse duration, the current residual time and the maximum EPSC amplitude increased. This is because with increased proton accumulation at the electrolyte/channel interface, the difference in the proton concentration in the electrolyte increased, which resulted in a longer relaxation time. These findings indicate that potato-starch EDL transistors can implement synaptic plasticity by modulating the channel conductivity.

**Figure 4:**
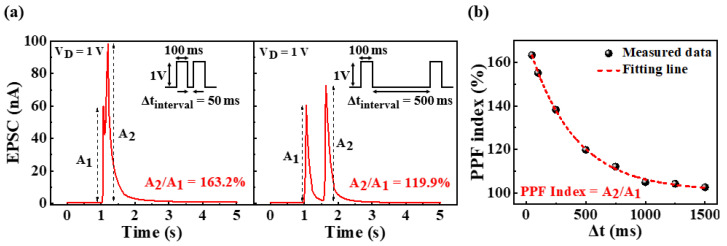
(**a**) Paired pulse facilitated EPSCs by two consecutive pre-synaptic spikes (1 V, 100 ms) at ∆t_interval_ = 50 ms and ∆t_interval_ = 500 ms and (**b**) paired-pulse facilitation (PPF) indices (A_2/_A_1_) plotted against pre-synaptic spike time interval (∆t_interval_) from 50 to 1500 ms.

In the biological nervous system, paired-pulse facilitation (PPF) exhibits short-term synaptic plasticity and is crucial to real-time decoding of temporal information in auditory or visual signals [32]. In a pair of consecutively applied pulses, the EPSC evoked by the spike increases when the second spike closely follows the previous one [33]. Figure 4a shows the paired-pulse-facilitated EPSC when applying two pulses with 1 V amplitude and 100 ms duration, with time intervals of 50 and 500 ms. In both cases, the second EPSC (A_2_) increased compared to the first EPSC (A_1_); however, the increase in A_2_ reduced at longer time intervals (Δt_interval_) between pulses. This was because of the relaxation time of the induced protons and the time interval between the two pulses. Upon the application of the first spike, the protons accumulated at the electrolyte/channel interface, resulting in a channel current. Following the pulse, the accumulated protons underwent a relaxation process from the interface over time. However, if Δt_interval_ was shorter than the complete relaxation time of the proton, the residual proton contributed to channel conduction, combined with proton accumulation by the second pulse [34]. Consequently, the longer the Δt_interval_ between pulses, the more fully relaxed the protons, thereby decreasing A_2_. The amplification factor of EPSCs was defined as the PPF index (A_2_/A_1_), and Figure 4b shows the measured PPF index for pulses with time intervals in the range of 50 to 1500 ms. A high PPF index of 163.2% was extracted for a short Δt_interval_ of 50 ms; however, this decreased with increase in the time interval between pulses, resulting in a PPF index of ~102% for a long Δt_interval_ of 1500 ms. Therefore, the second EPSC(A_2_) and the first EPSC(A_1_) were observed to became approximately equal with an increase in the time interval between pulses. These results indicate that the potato-starch EDL transistors can implement short-term synaptic plasticity. The PPF index was fitted using the following double-exponential decay relationship [35]:

(1) $$PPF\;index=A+C_{1}\exp(-\frac{\Delta t}{\tau_{1}})+C_{2}\exp(-\frac{\Delta t}{\tau_{2}})$$

where C_1_ and C_2_ represent the initial facilitation magnitudes and τ_1_ and τ_2_ are the characteristic relaxation times. The fitting results are plotted with dotted lines, where τ_1_ and τ_2_ are 381 and 383 ms, respectively, similar to the temporal scale of biological synapses. In addition, in many biological synapses, τ_1_ and τ_2_ exhibited similar values, and the distinction is unclear [35,36].

**Figure 5:**
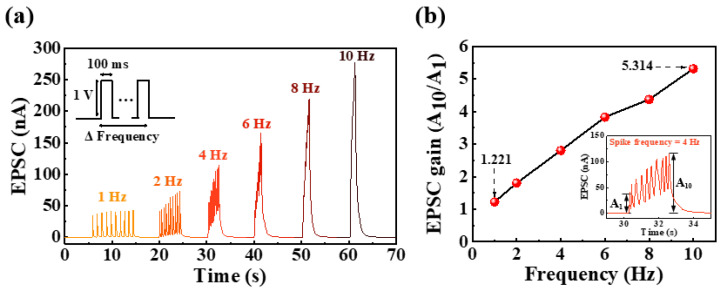
(**a**) EPSCs by applying ten consecutive pre-synaptic spikes with different frequencies and (**b**) EPSC gain (A_10/_A_1_) according to different applied spike frequencies.

Short-term synaptic facilitation is usable as a dynamic filter for signal transmission that depends on the frequency of stimulation in the biological neural network [36,37]. Specifically, synaptic facilitation contributes to high-pass time filtering [38]. In the prepared potato-starch EDL transistor, the PPF index increased with a decrease in the time interval between pulses, as shown in Figure 4b, which can be used as a high-pass time filter. To verify its function as a high-pass-time filter, the change in EPSC was examined through the application of 10 pulses with a duration and amplitude of 100 ms and 1 V, respectively, for a frequency range of 1 to 10 Hz. Figure 5a,b show the changes in EPSC with frequency and the EPSC gains (A10/A1), defined as the ratio of the 10th EPSC and the 1st EPSC by frequency, respectively. At a low frequency of 1 Hz, the EPSC gain was 1.22, and all EPSCs evoked by 10 spikes were not significantly increased compared with the first EPSC. In contrast, with an increase in the frequency, the EPSC gain gradually increased. Consequently, at a high frequency of 10 Hz, the EPSC gain increased to 5.32. This indicates that the proposed synaptic transistor can operate as a dynamic filter that mimics high-pass-time filtering via short-term facilitation.

**Figure 6:**
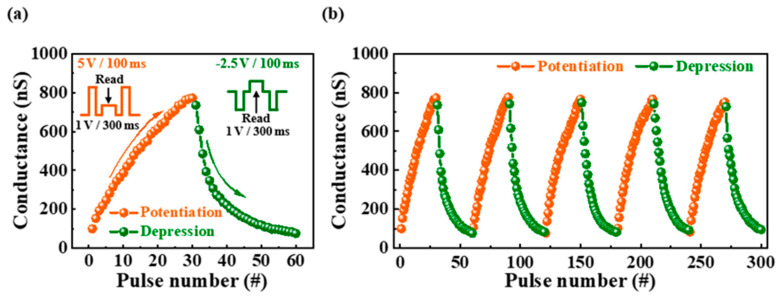
(**a**) Changes in channel conductance when 30 potentiation pulses and 30 depression pulses applied to pre-synapse and (**b**) endurance test of channel conductance modulation for potentiation/depression operation over 5 cycles.

In contrast to short-term plasticity, long-term plasticity represents a long-term change in synaptic weight, which increases with repeated and long-term application of stimuli. In long-term plasticity, an increase in synaptic weight implies a strengthening of the synaptic connection strength, which is referred to as long-term potentiation (LTP) [17,28]. However, a decrease in synaptic weight indicates a weakening of connection strength, which is referred to as long-term depression (LTD) [39,40]. Figure 6a shows the change in the channel conductance with the number of repetitive presynaptic stimulation pulses. The applied stimulus was divided into potentiation and depression pulses, which increase and decrease the conductance of the channel, respectively, as shown in the inset. As a presynaptic spike, 5 and −2.5 V potentiation and depression pulses, respectively, were successively applied to the gate 30 times for 100 ms. In addition, to read the change in channel conductance owing to stimulation, a pulse of 1 V was applied to the drain for 300 ms. Consequently, the channel conductance increased from 100.3 to 772.0 nS and decreased from 735.6 to 77.0 nS owing to the potentiation and depression pulses, respectively. Figure 6b shows the cycle test for conductance measured while repeating the potentiation/depression operation five times. Because the conductance change over five cycles was nearly constant, this endurance test provides reliability for changes in synaptic weights. Therefore, long-term plasticity, including LTP and LTD properties, induced by repeated stimulation was validated in potato-starch-electrolyte-based EDL synaptic transistors.

### 2.3. MNIST ANN Simulation

**Figure 7:**
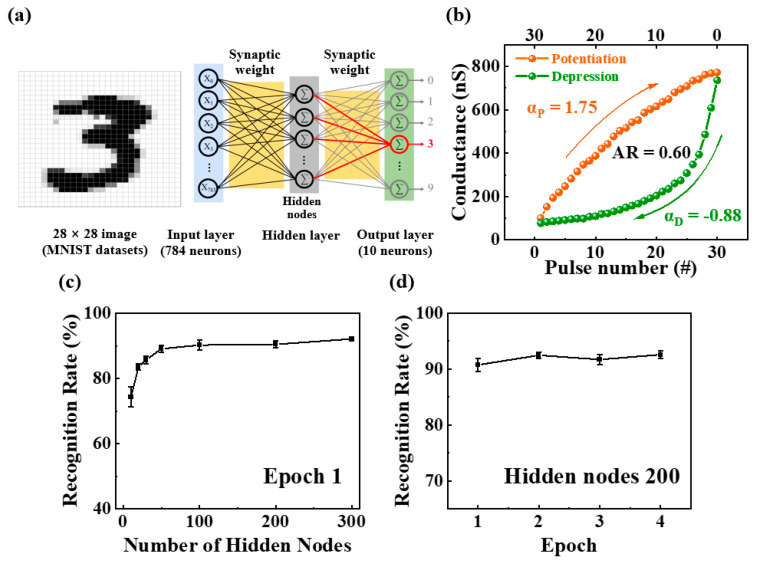
(**a**) Schematic of three-layer (input, hidden, and output layers) fully connected artificial neural network (ANN) for MNIST handwritten digit simulation; (**b**) nonlinearity analysis of normalized depression and potentiation (G/G_max_); recognition rate based on (**c**) number of hidden nodes in epoch 1 and (**d**) number of epochs with 200 hidden nodes.

To verify the viability of neuromorphic computing using the proposed synaptic transistor, we simulated the MNIST handwritten digit learning and recognition system using a three-layer perceptron network model. The designed artificial neural network (ANN) comprised input, hidden, and output layers, as shown in Figure 7a. The 784 and 20 neurons in the input and output layers represent 28 × 28 pixels of binarized MNIST data and digits from 0 to 9, respectively. As shown in Figure 7b, the nodes in each layer were connected to the nodes in the other layers through synapses. Synaptic weights, an indicator of the strength of the connection, were found to correlate with the normalized potentiation and depression conductance of the potato-starch electrolyte-based EDL synaptic transistors. Further, the normalized conductance was determined as the ratio of the measured conductance to the maximum conductance (G/G_max_). The dynamics of potentiation and depression, such as the dynamic range, asymmetric ratio (AR), and linearity, are critical to the accuracy of learning and recognition simulations. Here, the dynamic range implies the conductance modulation range defined as G_max_/G_min_, which was 10.03 for the fabricated potato-starch EDL transistors. Further, AR indicates the asymmetry of the changes in potentiation and depression conductance [40].

(2) $$AR=\frac{MAX\left|G_{p}\left(n\right)-G_{d}\left(n\right)\right|}{G_{p}\left(30\right)-G_{d}\left(30\right)}\;\mathrm{for}\;n=1\;to\;30$$

The closer AR is to 0, the closer it is to ideal learning, which was 0.6 for the proposed synaptic transistor. Because linearity in increasing and decreasing conductance is crucial to recognition simulations, the nonlinearity factor was extracted using the following equation [41]:

(3) G=GLRSα−GHRSα× w+GHRSα1αGHRS×(GLRS/GHRS)ω if α≠0, if α=0.

where ω is an internal variable (0–1) and G_max_ and G_min_ are the maximum and minimum conductances, respectively. 

The nonlinearity coefficient α was used to control the potentiation (α_p_) or depression (α_d_) with an ideal value of 1. The identified nonlinearity factors were 1.75 and −0.88 for potentiation (α_p_) and depression (α_d_), respectively. Normalized conductance and extracted factors were applied to the synaptic weights of the ANN. The designed ANN was trained using 60,000 MNIST training datasets, and recognition tests were performed using 10,000 additional datasets. Figure 7c shows the recognition rate obtained from the simulation when the number of neurons in the hidden layer was increased from 10 to 300. The recognition rate for 10 hidden nodes was 57%. It was observed that the recognition rate increased with the increase in hidden nodes, reaching 92% for 300 nodes. Figure 7d shows the recognition rate for the number of training epochs with 200 hidden nodes. Even after more epochs, the recognition rate reached a high level of 92% and remained almost constant. Therefore, these ANN simulation results demonstrate the potential of the proposed potato starch electrolyte-based EDL synaptic transistor as a promising artificial synapse for data-processing tasks such as pattern recognition.

## 3. Methods and Materials

### 3.1. Materials

The materials used in this study were photo inkjet printing paper (Hansol Paper., Ltd., Seoul, Republic of Korea), potato-starch powder (Hanseung Food Co., Ltd., Gimpo, Republic of Korea), acetic acid solution (purity > 99%, Sigma-Aldrich, Seoul, Republic of Korea), IGZO sputter target (In_2_O_3_:Ga_2_O_3_:ZnO = 4:2:4.1 mol%, THIFINE Co., Ltd., Incheon, Republic of Korea), and ITO sputter target (In_2_O_3_:SnO_2_ = 9:1 mol%, THIFINE Co., LTD., Incheon, Republic of Korea).

### 3.2. Fabrication Details of Potato-Starch EDL-Based Synaptic Transistors

**Figure 8:**
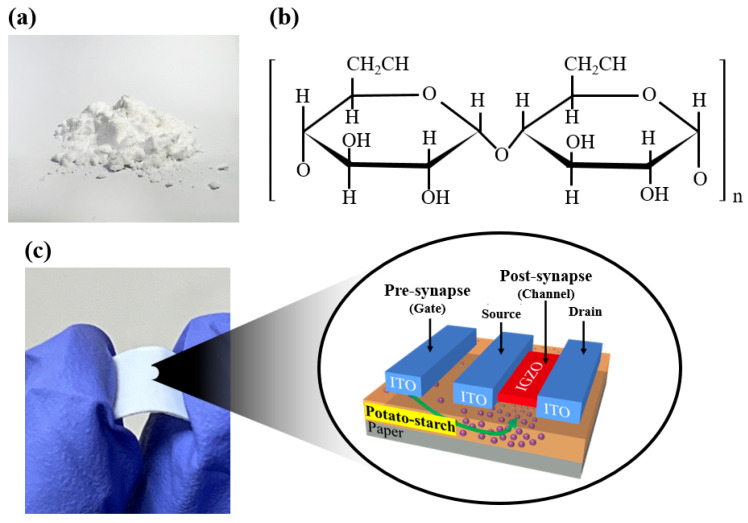
(**a**) Photographic image of potato-starch powder for electrolyte membrane formation of synaptic transistors; (**b**) molecular structure representing repeating units of potato starch. (**c**) Photographic image of the paper substrate on which the synaptic device is formed and a schematic diagram of the fabricated coplanar gate type artificial synaptic transistor.

The potato-starch solution was prepared by mixing 3 wt% of potato-starch powder, 0.5 wt% of acetic acid (purity > 99%), and 95.5 wt% of deionized water (DIW). The solution was mixed using a magnetic stirrer at 90 °C and 800 rpm for 20 min. Subsequently, it was filtered through a 5-μm pore size polytetrafluoroethylene syringe filter (Whatman International Ltd., Maidstone, UK) to remove particles. Figure 8a,b show photographic images of the potato-starch powder used to form the electrolyte membrane of the synaptic transistor and molecular structure of potato starch, respectively. Figure 8c shows a photographic image of the paper substrate on which the synaptic device was formed and a schematic diagram of the fabricated coplanar gated artificial synaptic transistor. 

The starting substrate, a photo inkjet printing paper, was blown with nitrogen gas to remove particles from the surface, and then the prepared potato-starch solution was coated onto the substrate using the drop-casting method and dried at room temperature in air for 24 h. An IGZO channel layer with a thickness of 50 nm was deposited on the solidified electrolyte membrane via RF magnetron sputtering at a working pressure, RF power, and Ar flow rate of 6 mTorr, 100 W, and 30 sccm, respectively. Subsequently, 150-nm-thick ITO source and drain (S/D) electrodes and coplanar gates were deposited by RF magnetron sputtering through a shadow mask, with working pressure, RF power, and Ar flow rates of 3 mTorr, 100 W, and 20 sccm, respectively. The sizes of the S/D and coplanar gate electrodes are 1000 × 200 μm, and the channel width (W) and length (L) are 1000 μm and 80 μm, respectively.

### 3.3. Characterization

The chemical groups and molecular structures of the potato-starch electrolyte membranes were analyzed using FTIR (IFS66v/s and Hyperion3000, Bruker Optiks, Billerica, MA, USA). The frequency-dependent capacitance of the potato-starch electrolyte-based capacitors was measured with a voltage level of 0.05 V, a current level of 100 μA, and a delay time of 0.0001 s using an Agilent 4284A precision LCR meter (Agilent Technologies, Santa Clara, CA, USA). Further, an Agilent 4156B semiconductor parameter analyzer was employed to measure the electrical properties and synaptic behavior of the potato starch-based synaptic transistors. In addition, the synaptic behavior was confirmed via the application of presynaptic spikes using an Agilent 8110A pulse generator. Moreover, all the behavioral characteristics of the synaptic devices were evaluated using a probe station in a dark box to minimize noise interference caused by external electrical signals and light.

## 4. Conclusions

This study fabricated artificial synaptic transistors with coplanar gate structures on paper substrates comprising biocompatible and low-cost potato-starch electrolyte gate insulators and indium–gallium–zinc oxide (IGZO) channels. The chemical groups and molecular structures of the potato-starch electrolyte membranes were analyzed using FTIR spectroscopy. Through C-*f* curve measurement with MIM (aluminum/potato-starch electrolyte/platinum) capacitors, the EDL effect of the potato-starch electrolyte was verified. Further, electrical characteristics evaluations of the coplanar gate-type potato-starch EDL transistors were performed through transfer and output curves, hysteresis window, and threshold voltage measurements. Synaptic functions were evaluated using single-spike EPSC, PPF, and frequency-dependent multi-spike EPSC. The single-spike EPSC and PPF characteristics mimic short-term synaptic plasticity, and high-pass time filtering in frequency-dependent EPSC mimics signal transmission in brain neural networks. Channel conductance modulation via repetitive presynaptic stimuli comprising potentiation and depression pulses enabled stable modulation of synaptic weights, thereby validating the long-term plasticity induced by repetitive stimuli. Finally, training and recognition simulations on the MNIST handwritten digit dataset were performed via multi-layer ANN, yielding a high recognition rate of 92% and found to efficiently mimic biological synapses. Therefore, the proposed potato starch electrolyte-based synaptic transistor can effectively mimic biological synapses using low-cost paper substrates and abundant organic electrolytes. Consequently, it is expected to be a building block for bio-friendly neuromorphic computing systems in the future.

## Data Availability

Not applicable.
